# The effects of nudges on purchases, food choice, and energy intake or content of purchases in real-life food purchasing environments: a systematic review and evidence synthesis

**DOI:** 10.1186/s12937-020-00623-y

**Published:** 2020-09-17

**Authors:** Marjolein C. Harbers, Joline W. J. Beulens, Femke Rutters, Femke de Boer, Marleen Gillebaart, Ivonne Sluijs, Yvonne T. van der Schouw

**Affiliations:** 1 Julius Center for Health Sciences and Primary Care, University Medical Center Utrecht, Utrecht University, P.O. box 85500, 3508 GA Utrecht, The Netherlands; 2 Department of Epidemiology and Data Science, Amsterdam Public Health Research Institute, Amsterdam UMC, location VUmc, Amsterdam, The Netherlands; 3 Department of Social Health and Organizational Psychology, Utrecht University, Utrecht, the Netherlands

**Keywords:** Nudging, Choice architecture, TIPPME, Socioeconomic position

## Abstract

**Background:**

Adults with a low socioeconomic position (SEP) are more likely to engage in unhealthy diets as compared to adults with high SEP. However, individual-level educational interventions aiming to improve food choices have shown limited effectiveness in adults with low SEP. Environmental-level interventions such as nudging strategies however, may be more likely to benefit low SEP groups. We aimed to review the evidence for the effectiveness of nudges as classified according to interventions in proximal physical micro-environments typology (TIPPME) to promote healthy purchases, food choice, or affecting energy intake or content of purchases, within real-life food purchasing environments. Second, we aimed to investigate the potentially moderating role of SEP.

**Methods:**

We systematically searched PubMed, EMBASE, and PsycINFO until 31 January 2018. Studies were considered eligible for inclusion when they i) complied with TIPPME intervention definitions; ii) studied actual purchases, food choice, or energy intake or content of purchases, iii) and were situated in real-life food purchasing environments. Risk of bias was assessed using a quality assessment tool and evidence was synthesized using harvest plots.

**Results:**

From the 9210 references identified, 75 studies were included. Studies were generally of weak to moderate quality. The most frequently studied nudges were information (56%), mixed (24%), and position nudges (13%). Harvest plots showed modest tendencies towards beneficial effects on outcomes for information and position nudges. Less evidence was available for other TIPPME nudging interventions for which the harvest plots did not show compelling patterns. Only six studies evaluated the effects of nudges across levels of SEP (e.g., educational level, food security status, job type). Although there were some indications that nudges were more effective in low SEP groups, the limited amount of evidence and different proxies of SEP used warrant caution in the interpretation of findings.

**Conclusions:**

Information and position nudges may contribute to improving population dietary behaviours. Evidence investigating the moderating role of SEP was limited, although some studies reported greater effects in low SEP subgroups. We conclude that more high-quality studies obtaining detailed data on participant’s SEP are needed.

**Registration:**

This systematic review is registered in the PROSPERO database (CRD42018086983).

## Introduction

An unhealthy diet is one of the major risk factors for non-communicable diseases (NCDs), such as type 2 diabetes and cardiovascular disease [1]. Adults with a low socioeconomic position (SEP) in particular are at high risk for NCDs, as they are more likely to engage in unhealthy diets as compared to adults with high SEP [2]. Despite this, individual-level educational interventions that aim to improve healthy food choices have shown to have limited effectiveness in adults with low SEP and may increase health inequalities [3]. This may partly be attributed to the fact that these interventions often necessitate access to various resources (e.g., knowledge, skills, social networks) which may be more limited in low SEP groups [4, 5]. Alternatively, environmental-level interventions are more likely to benefit adults with low SEP and reduce health inequalities [3], because they rely to a lesser extent on an individual’s access to resources but rather create healthy opportunities for all.

The rationale underlying such environmental-level interventions is rooted in dual process models of human behaviour, which conceptualize the regulation of human behaviour into two main cognitive processes: 1) an unconscious, fast, and automatic cognitive process, and 2) a conscious, slow, and more effortful cognitive process [6]. Whereas individual-level educational interventions tap into the conscious and effortful processes – by for example providing nutrition knowledge to target populations – environmental interventions make use of environmental cues or heuristics that subconsciously guide food-decision making [7], thus requiring limited amounts of cognitive resources.

Nudging has been proposed as a promising environmental intervention strategy for modifying food choices. The term ‘nudge’ was originally coined by Thaler and Sunstein in 2008 and defined as: ‘*Any aspect of the choice architecture that alters people’s behaviour in a predictable way, without forbidding any options or significantly changing their economic incentives’ (p.6*) [8]. Nudging became popular as it opposed the reigning idea that humans are rational actors who constantly seek opportunities that maximize their utility. Instead, it acknowledges that people’s ability to make rational decisions is limited by cognitive boundaries, biases and habits, leading people to make choices not compatible with their long-term goals [9]. Nudges make use of the same principles that cause flawed decision-making, to steer people towards choices that serve them in their own interest. When applied to modifying diets, this means that nudges make healthy choices more easy, by for example making them more salient, without constraining choice for unhealthy alternatives [9].

So far, numerous nudging studies have been performed describing a wide range of interventions, for example placing healthier foods at convenient and visible locations in supermarkets (e.g., position nudge) or making healthy foods salient through the use of signage (e.g., information nudge). To establish more conceptual clarity regarding nudging interventions and to facilitate evidence synthesis, the typology of interventions in proximal physical micro-environments (TIPPME) was introduced, distinguishing six distinct nudging interventions types: availability, position, functionality, presentation, size, and information [10].

The multiple systematic reviews and meta-analyses on the effectiveness of TIPPME nudging interventions in modifying food choices or consumption [11–13] mainly focused on availability and position nudges [12, 13] or specific foods [11], and studies were primarily conducted in laboratory settings. Only one of these systematic review addressed the question whether the effects of nudging interventions are moderated by SEP, for which indications were found [13]. Therefore, insights are lacking on the effectiveness of other TIPPME intervention types in real-life food purchasing environments, and the moderating role of SEP.

In the present systematic review, our first aim is to review the evidence for the effectiveness of nudges as classified according to the TIPPME typology in promoting healthy purchases, food choice, or affecting energy intake or content of purchases within real-life food purchasing environments among adult populations. Second, we aimed to investigate the potentially moderating role of SEP.

## Methods

The protocol for the present systematic review was registered in the PROSPERO database (registration number: CRD42018086983). A systematic literature search was conducted in accordance with the guidelines in the Reporting Items for Systematic Reviews and Meta-Analysis (PRISMA) statement (www.prisma-statement.org) (Additional file 1).

### Data sources and searches

In order to maximize the yield of our search, we adopted an elaborate search strategy including general nudging terms (e.g., nudging and choice architecture) as well as more specific nudging terms (e.g., signage) according the TIPPME typology (Table 1). Types of nudges considered in other categorizations were evaluated on their applicability to the current review [14, 15]. As a result, the search strategy was further extended by adding the default nudge, which we defined as follows: ‘to provide a standard food option for which no active choice needs to be made’.

**Table 1:** Overview of nudging interventions in TIPPME as defined by Hollands et al. [10]

Intervention type	Definition
Availability	To add or remove (some or all) products or objects to increase, decrease, or alter their range, variety, or number
Position	To alter the position, proximity, or accessibility of products or objects
Functionality	To alter the functionality or design of products or objects to change how they work, or guide or constrain how people use or physically interact with them
Presentation	To alter visual, tactile, auditory or olfactory properties of products, objects or stimuli
Size	To alter size or shape of products or objects
Information	Add, remove, or change words, symbols, numbers or pictures that convey information about the product or object or its use

For the search queries, search terms for the (type of) nudging intervention, outcome, and setting were combined using Boolean operators and were limited to title and abstract. The search strategies for each of the databases can be found in Additional file 2. We systematically searched the databases PubMed, EMBASE, and PsycINFO until 31 January 2018. Additionally, references included in existing reviews were included for screening [11, 12, 16].

### Study selection

Titles, abstracts, and full-text articles retrieved from database searches were screened for eligibility in duplicate by a team of five researchers (MH, FdB, IS, JWJB, FR). Studies were included if they: 1) involved a manipulation of the food purchasing environment, in such a way that the availability, position, functionality, presentation, size, and/or information of products (e.g., foods), related objects (e.g., shelfs), or the wider environment (e.g., supermarket) was altered; 2) examined the effects on actual food purchases, energy intake or energy content of purchases, or food choice; 3) were situated in a food purchasing environment where people purchase food or meals on a regular basis; 4) were conducted among adult populations; 5) were originally published articles and were written in English language.

Studies were excluded if they: 1) did not report the effects of the nudges separately from other non-nudge interventions, such as pricing interventions; 2) studied the effects of nudges on behavioural intent; 3) were performed in settings in which people do not purchase food or meals on a regular basis (e.g., sit-down restaurants); 4) changed the intrinsic characteristics of foods (e.g., dietary composition); 5) examined the effects of mandatory legislation.

Inconsistencies in eligibility judgements were resolved by discussion among two reviewers (MH and IS) and if consensus could not be reached, inconsistencies were resolved by discussion with a third reviewer (JWJB, FR, or FdB). After this process was completed, titles, abstracts, and full-text articles retrieved from the reference lists of existing reviews were screened for eligibility by MH. A 10% subsample of the studies retrieved from the reference lists was checked by a second reviewer (IS), which revealed no inconsistencies in eligibility judgements.

### Quality assessment

Risk of bias was assessed using the Quality Assessment Tool for Quantitative Studies [17], as this tool was specifically designed to critically appraise public health interventions and encompassed a wide range of research designs, including non-randomized designs. This tool evaluates the risk of bias with regard to selection of study participants, study design, confounding variables, blinding, data collection methods, and withdrawals and drop-outs. Each domain can be attributed a weak, moderate or strong quality score. Articles were considered of i) strong quality if no domains were rated as weak; ii) moderate quality if only one domain was rated as weak; 3) weak if at least two domains were rated as weak. Quality assessment was conducted in duplicate by a team of five researchers (MH, FdB, IS, JWJB, FR). Inconsistencies were resolved by discussion with a third reviewer.

### Data extraction

Data extraction was performed by one researcher (MH) using a predefined data extraction form, and conducted in duplicate for a subsample of the included studies (*n* = 8), which showed high levels of agreement. Data was extracted on the type of nudge (including nudge description), country, study design, study size, intervention duration, SEP, setting, study outcomes, outcome assessment, and main findings.

### Data synthesis

For the tabulation of study characteristics and main findings, nudges were classified using the TIPPME intervention typology (MH & FdB) into either one of the following intervention types: availability, position, functionality, presentation, size or information. On the basis of the quality assessment, study design was categorized into before-after studies (both within- and between-subjects), controlled trials, or randomized controlled trials. Intervention duration was defined as the duration for which the nudge was implemented and categorized according to the following categories: ≤ 1 week; > 1 week & ≤ 1 month; 1 < month(s) ≤ 6; 6 < months ≤12 and > 1 year. Study size could pertain to amount of purchases and/or transactions, number of customers, or number of stores. Study outcomes could pertain to purchases, energy intake or energy content of purchases or food choice. Outcome assessment was categorized as either one or a combination of the following: point-of-sale system, observer-reported, computer-generated response, digital photographic method, food weighing, hand counts, questionnaires, dietary recall, and records of inventory movement. Lastly, we report SEP characteristics for each study based on descriptive characteristics for proxies of SEP reported in the baseline table or in-text (e.g., educational level, job type).

Besides the tabulation of study characteristics and main findings, we visualized the main findings and study characteristics of studies within each of the TIPPME categories in harvest plots [18]. The harvest plot groups studies according to their intervention effect (positive/negative or no effect) in a matrix, and allows to further incorporate relevant study information by varying characteristics of the matrix, including bar length, width, and colour, and by adding rows to the matrix. As such, harvest plots provide a qualitative summary to the reader by enabling them to visually appraise the most prominent patterns in the matrix, and judge study characteristics and study quality.

For the present review, the matrix comprises three columns representing the intervention effect (increase, no change, or decrease) and three rows comprising the types of outcomes (purchases, energy intake or energy content of purchases or food choice). Studies were plotted in the matrix based on the direction of the association that was reported for each outcome (e.g., if a nudge is associated with higher purchases, this study was plotted in the ‘increase’ column). Each study was plotted in the matrix using bars, with a study reference number below the bar corresponding to the tabulation of the study characteristics and main findings in Table 2. If studies assessed multiple outcomes, studies appear in the matrix for each outcome denoted by an additional letter (e.g., 1a, 1b). The bars were further modified to represent several relevant study characteristics. More specifically, high bars represent RCTs and controlled trials and low bars represent before-after study designs; narrow bars indicate shorter study duration and increasing width indicates longer study duration; red bars indicate unhealthy foods, blue bars indicate healthy foods, and white bars indicate calorie intake or content of purchases. Lastly, settings as retrieved from the data extraction were categorized into cafeterias (denoted by letter C) and supermarkets and small food stores (denoted by letter S).

**Table 2:** Study characteristics and main findings of included studies categorized by TIPPME intervention type

Author	Year	Country	Nudge description	Study design	Study size	Intervention duration	Setting	SEP	Study outcome(s)	Outcome assessment	Main finding(s)	Quality assessment
*Information nudges (symbols)*												
Cawley et al. [19]	2015	USA	Supermarket items were assigned with stars indicating their relative healthiness	Pre-post	168 supermarkets	> 1 year	Supermarket	N/A	Purchases healthy items (any stars); Purchases of unhealthy items (no stars)	Point of sale system	1a. Purchases of healthy items were not affected 1b. Purchases of unhealthy items decreased^a^	Moderate
Dubbert et al. [20]	1984	USA	Labels indicating low-calorie choices were placed besides serving location	Pre-post	6970 customers	> 1 week & ≤ 1 month	Cafeteria	N/A	Purchases of vegetables; Purchases of salad; Purchases of entrees; Caloric content of meal purchased	Point of sale system and observer reported	2a. Increased vegetable purchases^a^ 2b. Increased salad purchases^a^ 2c. Entrée purchases not affected 2d. Caloric content of meals purchased not affected	Weak
Elbel et al. [21]	2013	USA	Unhealthy items were assigned a tag stating ‘less healthy’	Pre-post	3680 purchases	> 1 week & ≤ 1 month	Small food store	Store catered to low-income, minority and immigrant population	Purchases of healthy items; Caloric content of purchases	Point of sale system	3a. Probability of purchasing healthy items increased^a^ 3b. Caloric content of items purchased decreased^a^	Strong
Eldridge et al. [22]	1997	USA	Menu boards indicated healthy items with a green check-mark	Pre-post	7 cafeterias	6 < months ≤12	Cafeteria	N/A	Purchases of all targeted items	Point of sale system	4. Purchases of targeted items were not affected	Moderate
Freedman et al. [23]	2011	USA	Healthy foods were identified with a promotional logo on shelf-tags	Pre-post	1 small food store	1 < month(s) ≤ 6	Small food store	N/A	Purchases of all targeted items	Point of sale system	5. Purchases of targeted items were not affected	Moderate
Hobin et al. [24]	2017	Canada	Supermarket items were assigned with stars indicating their relative healthiness	CT	44 intervention supermarkets; 82 control supermarkets	6 < months ≤12	Supermarket	Supermarkets were located in area where 13.1% had no secondary school diploma	Purchases of healthy items; Caloric content of purchases	Point of sale system	6a. Average mean star rating per product purchased increased, so healthy purchases increased^a^ 6b. Caloric content of purchases was not affected	Strong
Hoefkens et al. [25]	2011	Belgium	Healthy meal suggestions were assigned with stars (0–3 stars)	Pre-post	224 customers	> 1 week & ≤ 1 month	Cafeteria	N/A	Meal choice (0–3 stars) and energy intake	Questionnaire	7a. Meal choice was not affected; 7b. Energy intake was not affected	Weak
Johnson et al. [26]	1990	USA	Labels indicating low-calorie choices were placed besides serving location	Pre-post	413 customers	> 1 week & ≤ 1 month	Cafeteria	N/A	Caloric content of purchases	Observer reported	8. Caloric content of purchases was not affected	Weak
Lassen et al. [27]	2014	Norway	Healthy choices were labelled with the Keyhole symbol	CT	270 customers	6 < months ≤12	Cafeteria	59% employed as office and administrative personnel or as technical staff	Energy density of consumed foods	Digital photographic method	9. Energy density decreased^a^	Strong
Levin et al. [28]	1996	USA	Low-fat entrees were labelled with a heart-shaped symbol	CT	2 cafeterias	6 < months ≤12	Cafeteria	N/A	Purchases of targeted items	Point of sale system	10. Purchases of targeted items increased^a^	Moderate
Sproul et al. [29]	2003	USA	Healthy entrees were labelled with a promotional logo, which additionally provided nutritional information	Pre-post	1 cafeteria	1 < month(s) ≤ 6	Cafeteria	N/A	Purchases of targeted entrees	Point of sale system	11. Purchases of targeted entrees were not affected	Moderate
Sutherland et al. [30]	2010	USA	Supermarket items were assigned with stars indicating their relative healthiness	Pre-post	168 supermarkets	> 1 year	Supermarket	N/A	Purchases of star-labelled items	Point of sale system	12. Purchases of star-labelled items increased^a^	Moderate
Vyth et al. [31]	2011	The Netherlands	Healthy sandwiches, soups, and fresh fruit were identified with a promotional logo	RCT	13 intervention cafeterias; 12 control cafeterias	> 1 week & ≤ 1 month	Cafeteria	N/A	Purchases of healthy sandwiches; Purchases of healthy soups; Purchases of fresh fruit	Point of sale system	13a. Purchases of healthy sandwiches were not affected 13b. Purchases of healthy soups were not affected 13c. Fruit purchases increased^a^	Moderate
Mazza et al. [32]	2017	USA	Emoticons highlighted healthy items	Pre-post	1 cafeteria	1 < month(s) ≤ 6	Cafeteria	N/A	Purchases of healthy beverages Purchases of healthy chips	Point-of-sale system	14a. Purchases of healthy chips were not affected 14b. Purchases of healthy beverages were not affected	Moderate
Steenhuis et al. [33]	2004	The Netherlands	In the labelling program, low-fat products were identified with a promotional logo.	RCT	17 cafeterias were randomly assigned to either of 4 conditions (including control and labelling program)	1 < month(s) ≤ 6	Worksite cafeteria	2% low educational level	Purchases of low-fat items (milk, butter, cheese, meat, desserts).	Point of sale system and questionnaire	15a. Purchases of low-fat desserts increased^a^ 15b. Purchases of milk were not affected 15c. Purchases of butter were not affected 15d. Purchases of cheese were not affected 15e. Purchases of meat were not affected	Weak
*Information nudges (nutrition information)*												
Cioffi et al. [34]	2015	USA	Nutrition labels were added to a selection of pre-packaged meals and snacks	Pre-post	20 small food stores	6 < months ≤12	Small food store	N/A	Purchases of low calorie foods; Purchases of high calorie foods; Caloric content of purchases	Point of sale system	1a. Purchases of low calorie foods increased^a^ 1b. Purchases of high calorie foods decreased 1c. Caloric content of items purchased decreased^a^	Moderate
Hammond et al. [35]	2015	Canada	Calorie labels were added to all cafeteria menu boards and food stations	Pre-post	159 customers	≤ 1 week	University cafeteria	N/A	Caloric content of purchases; Calories consumed	Questionnaire	2a. Caloric content of purchases decreased^a^ 2b. Calorie intake decreased^a^	Weak
Milich et al. [36]	1976	USA	Foods were labelled with their caloric value	Pre-post	450 customers	≤ 1 week	Hospital cafeteria	N/A	Caloric content of purchases	Observer reported	3. Caloric content of purchases decreased; (*p* = 0.06)	Weak
Vanderlee et al. [37]	2014	Canada	Energy, sodium and fat content were displayed on digital menu boards, as well as a health logo for healthier items	CT	497 customers at intervention site; 506 customers at control site	1 < month(s) ≤ 6	Hospital cafeteria	14% low educational level (high school or less) 15% low income (<$CAN 40000)	Calorie intake	Questionnaire	4. Caloric intake decreased^a^	Weak
Aron et al. [38]	1995	UK	Foods were provided with nutrition labels	CT	65 intervention customers; 35 control customers	≤ 1 week	University cafeteria	N/A	Calorie intake	Questionnaire	5. Caloric intake increased^a^	Weak
Chu et al. [39]	2009	USA	Simplified nutrition labels were posted at the point of selection for entrée dishes	Pre-post	1 cafeteria	> 1 week & ≤ 1 month	University cafeteria	N/A	Caloric content of purchases	Point of sale system	6. Caloric content of purchases was not affected	Moderate
Webb et al. [40]	2011	USA	Calorie information was posted on menu boards or was provided only on posters placed away from the point of decision.	CT	1 experimental cafeteria; 1 control cafeteria	1 < month(s) ≤ 6	Hospital cafeteria	13% low educational level (< eighth grade, some high school and high school graduate)	Purchases of healthy side dishes; Purchases of healthy snacks; Purchases of healthy entrees	Point of sale system	7a. Purchases of sides dishes increased^a^ 7b. Purchases of snacks increased^a^ 7c. Purchases of entrees were not affected	Moderate
Chen et al. [41]	2017	Taiwan	Entrees and side dishes were labeled with traffic-light labels	Pre-post	276 customers for first survey; 205 customers for second survey	6 < months ≤12	Worksite cafeteria	N/A	Choice for green-labelled food; Attempt to avoid red-labelled food	Questionnaire	8a. Choice for green entrée increased^a^; 8b. Attempt to avoid red-coloured items was not affected.	Moderate
Sonnenberg et al. [42]	2013	USA	Food and beverages were labelled red, yellow, or green on either the menu board, shelf, or directly on the packaging.	Pre-post	389 customers	1 < month(s) ≤ 6	Hospital cafeteria	N/A	Purchases of green items Purchases of red items	Point of sale system	9a. Healthy (green) item purchases were not affected 9b. Unhealthy (red) item purchases were not affected	Strong
Whitt et al. [43]	2017	USA	Items were labelled green (healthy), yellow (neutral) or red (unhealthy).	Pre-post	1 small food store	1 < month(s) ≤ 6	Small food store	N/A	Purchases of green items Purchases of red items	Point of sale system	10a. Purchases of healthy (green) items increased^a^ 10b. Purchases of unhealthy (red) items decreased^a^	Moderate
*Information nudges (signage)*												
Allan et al. [44]	2015	UK	Signs visually arranged snacks and drinks from least caloric to most caloric, with arrows indicating their location in store	RCT	> 20,000 purchases	1 < month(s) ≤ 6	Small food store	N/A	Purchases of high-calorie snacks Purchases high-calorie drinks	Point of sale system	1a. Purchases of high calorie snacks decreased^a^; 1b. Purchases of high calorie drinks were not affected.	Moderate
Buscher et al. [45], study 1	2001	Canada	Signs with promotional prompts were located at the cafeteria entrance and in front of the targeted foods	Pre-post	2280 students potentially exposed to the intervention	> 1 week & ≤ 1 month	University cafeteria	N/A	Purchases of vegetable basket Purchases of pretzels Purchases of yoghurt Purchases of fruit basket	Point of sale system and hand-counts	2a. Vegetable basket purchases were not affected 2b. Pretzel purchases increased^a^ 2c. Yoghurt purchases increased^a^ 2d. Fruit basket purchases were not affected	Moderate
Buscher et al. [45], study 2	2001	Canada	Signs with promotional prompts were located at the cafeteria entrance and in front of the targeted yoghurt	Pre-post	2280 students potentially exposed to the intervention	> 1 week & ≤ 1 month	University cafeteria	N/A	Purchases of yoghurt	Point of sale system	3. Yoghurt purchases increased^a^	Moderate
Montuclard et al. [46]	2017	USA	A water sign was taped to the cafeterias soda dispensers and coffee dispensers	Pre-post	357 students pre-intervention survey; 301 students post-intervention survey	1 < month(s) ≤ 6	University cafeteria	N/A	Choice for water	Questionnaire	4. Choice for water increased^a^	Moderate
Ogawa et al. [47]	2011	Japan	Health and nutrition information related to consumption of fruits and vegetables was displayed on posters near fruit/vegetable display and/or checkout counter	CT	1 intervention supermarket; 1 control supermarket	1 < month(s) ≤ 6	Supermarket	N/A	Purchases of fruits Purchases of vegetables	Point of sale system	5a. Vegetable purchases increased^a^ 5b. Fruit purchases were not affected.	Moderate
Policastro et al. [48]	2017	USA	Water consumption was promoted through signage promoting swapping soda for water	Pre-post	2393 students covering 6730 transactions	≤ 1 week	University cafeteria	N/A	Choice for water	Point of sale system	6. Water purchases increased^a^	Moderate
Scourboutakos et al. [49]	2017	Canada	Posters promoted water and fruit and vegetable consumption	Pre-post	368 to 510 students per data collection day	1 < month(s) ≤ 6	University cafeteria	N/A	Purchases of water Purchases of fruits Purchases of vegetables	Observer reported	7a. Purchases of water increased^a^ 7b. Purchases of fruit increased^a^ 7c. Purchases of vegetables increased^a^	Weak
Mazza et al. [32]	2018	USA	A health message stating the % of daily calories contained in beverages, and required exercise to burn calories of chips	Pre-post	1 cafeteria	> 1 week & ≤ 1 month	Hospital cafeteria	N/A	Purchases of healthy chips Purchases of healthy beverages	Point of sale system	8a. Purchases of healthy chips were not affected 8b. Purchases of healthy beverages were not affected	Moderate
Mazza et al. [32]	2018	USA	A health message stating the % of daily calories contained in chips, and required exercise to burn calories of beverages	Pre-post	1 cafeteria	> 1 week & ≤ 1 month	Hospital cafeteria	N/A	Purchases of healthy chips Purchases of healthy beverages	Point of sale system	9a. Purchases of healthy chips increased^a^ 9b. Purchases of healthy beverages were not affected	Moderate
Payne et al. [50], study 1	2015	USA	Messages on grocery carts stated the number of fruits and vegetable items customers of that particular store normally purchased	CT	396,017 individual person transactions	> 1 week & ≤ 1 month	Supermarket	Supermarkets were located in area with 7% unemployment and 24% only high school education	Purchases of fruits and vegetables	Point-of-sale system	10. Fruit and vegetable purchases increased^a^	Strong
Payne et al. [50], study 2	2015	USA	Messages on grocery carts stated the number of F&V items customers of that particular store normally purchased	Pre-post	575,689 individual person transactions	> 1 week & ≤ 1 month	Supermarket	Supermarkets were located in area with 7% unemployment and 24% only high school education	Purchases of fruits and vegetables	Point-of-sale system	11. Fruit and vegetable purchases increased^a^	Strong
Salmon et al. [51]	2015	The Netherlands	The presence of a banner with was manipulated (absent/present, which stated that a particular low-fat cheese was the most sold brand of cheese in the supermarket.	CT	127 customers	N/A	Supermarket	10% low educational level (primary school or lower levels of high school)	Purchases of low-fat cheese	Collection of receipts	12. Purchases of low-fat cheese were not affected	Strong
*Position nudges*												
Kroese et al. [52]	2016	The Netherlands	Unhealthy snacks at the check-out counter were replaced by healthy snacks	CT	2 intervention stores; 1 control store	≤ 1 week	Small food store	N/A	Purchases of healthy snacks; Purchases of unhealthy snacks	Point-of-sale system	1a. Healthy snack purchases increased^a^ 1b. Unhealthy snack purchases were not affected	Moderate
Meiselman et al. [53], study 1	1994	UK	Candy was repositioned from four cash-points to one distant cash-point	Pre-post	43 students	≤ 1 week	University cafeteria	N/A	Choice for candy Energy intake	Questionnaire and food weighing	2a. Candy selection decreased^a^ 2b. Energy intake was not affected	Weak
Meiselman et al. [53], study 2	1994	UK	Potato chips were repositioned from meal line to distant snack bar	Pre-post	60 students	> 1 week & ≤ 1 month	University cafeteria	N/A	Choice for potato chips	Questionnaire	3. Potato chips selection decreased^a^	Weak
Meyers et al. [54]	1980	USA	High calorie desserts were placed in the rear position on buffet line	Pre-post	4412 food choices were observed	≤ 1 week	Hospital cafeteria	N/A	Choice for high calorie desserts	Observer-reported	4. High calorie dessert choice was not affected	Moderate
Rozin et al. [55], study 3	2011	USA	Salad bar ingredients were placed on edge position of salad bar vs. middle position	Pre-post	1 cafeteria	1 < month(s) ≤ 6	Hospital cafeteria	N/A	Purchases of salad bar ingredients	Food weighing	5. Salad bar purchases increased^a^	Moderate
Van Gestel et al. [56]	2017	The Netherlands	Unhealthy snacks at the check-out counter were replaced by healthy snacks	Pre-post	1 small food store	> 1 week & ≤ 1 month	Small food store	N/A	Purchases of healthy snacks	Point of sale system	6. Healthy snack purchases increased^a^	Moderate
Chapman et al. [57], study 1	2012	UK	Confectionery was removed from check-out counters and replaced by fruit	Pre-post	1 cafeteria	≤ 1 week	University cafeteria	N/A	Purchases of fruit; Purchases of confectionery	Point of sale system	7a. Healthy fruit purchases decreased^a^ 7b. Unhealthy confectionary purchases increased^a^	Moderate
De Wijk et al. [58]	2016	The Netherlands	Wholegrain bread was placed near entrance vs. away from entrance	CT	2 supermarkets	1 < month(s) ≤ 6	Supermarket	N/A	Purchases of wholegrain bread	Point of sale system	8. Healthy bread purchases were not affected	Moderate
Thorndike et al. [59]	2017	USA	Stores improved visibility of fruits and vegetables through new supplies (e.g., baskets, shelving)	RCT	3 intervention stores; 3 control stores	1 < month(s) ≤ 6	Small food store	Store was located in low-income urban community	Purchases of fruits and vegetables	Point of sale system (WIC voucher redemption)	9. Fruit and vegetable purchases increased^a^	Moderate
Winkler et al. [60]	2016	Denmark	Sugar confectionery at one checkout counter was replaced by healthy snacks	CT	4 intervention stores; 2 control stores	> 1 week & ≤ 1 month	Supermarket	N/A	Purchases of healthy snacks; Purchases of sugar confectionary	Point of sale system	10a. Healthy snack purchases were generally not affected 10b. Unhealthy purchases were not affected	Moderate
*Mixed nudges*												
Gittelsohn et al. [61]	2013	USA	Environmental changes included demonstrations of healthier cooking methods, taste-tests, and display of point-of-purchase materials (e.g., posters and shelf labels)	RCT	98 participants from intervention condition; 47 participants from control condition	> 1 year	Supermarket	Years of schooling; 10.9y (intervention particpants) and 9.3y (control participants)	Healthy food purchasing score Unhealthy food purchasing score	Questionnaire	1a. Healthy food purchasing score was not affected 1b. Unhealthy food purchasing score was not affected	Weak
Dorresteijn et al. [62]	2013	The Netherlands	Environmental changes included signage promoting low-sodium soup and low-fat croissants. Also, margarine was made less accessible whereas butter was made more accessible.	Pre-post	1 cafeteria	> 1 week & ≤ 1 month	Hospital cafeteria	N/A	Purchases of normal soup Purchases of healthier soup Purchases of normal croissants Purchases of healthier croissants Purchases of butter Purchases of margarine	Point of sale system and hand-counts	2a. Normal soup purchases were not affected 2b. Healthier soup purchases were not affected 2c. Normal croissant purchases were not affected 2d. Healthier croissant purchases were not affected 2e. Butter purchases increased^a^ 2 f. Margarine purchases decreased^a^	Weak
Gamburzew et al. [63]	2016	France	Environmental changes included shelf labels indicating healthy foods, signage explaining the labelling system, placement strategies, and a taste-testing booth.	CT	6625 customers	1 < month(s) ≤ 6	Supermarket	N/A	Purchases of targeted foods (fruits and vegetables; starches; meat/fish/eggs; mixed dishes and sandwiches; dairy products).	Point of sale system	3a. Purchases of fruits and vegetables increased^a^ 3b. Purchases of starches increased^a^ 3c. Purchases of meat/fish/eggs were not affected 3d. Purchases of mixed dishes/sandwiches were not affected 3e. Purchases of dairy were not affected	Weak
Gittelsohn et al. [64]	2010	USA	Environmental changes included posters, shelf labels, cooking demonstrations and taste tests.	CT	64 intervention participants; 53 control participants	6 < months ≤12	Supermarket	For intervention and comparison group, respectively: Years of schooling, 12.5y and 12.4y; Percentage unemployed, 35.9 and 18.8%.	Healthy food purchasing score Unhealthy food purchasing score Calorie intake	Questionnaires and dietary recall	4a. Healthy food purchasing score was not affected 4b. Unhealthy food purchasing score was not affected 4c. Calorie intake was not affected	Strong
Foster et al. [65]	2014	USA	Environmental changes included placement strategies (multiple facings, prime placement), signage, shelf-tags, cross-promotion of healthy foods, and taste testing.	RCT	4 intervention supermarkets; 4 control supermarkets	6 < months ≤12	Supermarket	Supermarkets located in low-income, high-minority neighborhoods	Purchases of targeted foods (milk, cereal, frozen meals, in aisle-beverages, checkout cooler beverages, water)	Point of sale system	5a. Purchases of some targeted products within the milk category increased^a^ 5b. Purchases of cereals were not affected 5c. Purchases of some targeted products within the frozen meal category increased^a^ 5d. Purchases of some targeted products within the in-aisle beverages category decreased to a lesser extent in the intervention stores as compared to control^a^ 5e. Purchases of check-out cooler beverages were not affected 5 f. Purchases of water increased^a^	Moderate
Lawman et al. [66]	2015	USA	Availability of healthy foods was increased and promoted through banners, shelf labels, and recipes. A subset of stores was provided additional business trainings and mini-grants for storing their inventory of healthy foods (high-intensity intervention).	Pre-post	8671 customers at baseline; 5949 customers at follow-up	6 < months ≤12	Small food store	N/A	Mean energy purchased	Observer reported	6. The intervention did not affect mean energy purchased.	Moderate
Levy et al. [67]	2012	USA	Foods were labelled green, orange or red. Additionally, a choice architecture intervention was added which increased visibility and accessibility of green-labelled foods and beverages while decreasing the same for certain red-labelled items.	Pre-post	4642 customers	1 < month(s) ≤ 6	Hospital cafeteria	28% employed as service workers, administrative support, technicians	Purchases of green items Purchases of red items	Point of sale system	7a. Green item purchases increased^∞^ 7b. Red item purchases decreased^∞^	Strong
Lowe et al. [68]	2010	USA	Environmental changes included increased availability of foods lower in energy density. Additionally, a labelling system was introduced which color-coded food items.	Pre-post	49 customers	1 < month(s) ≤ 6	Hospital cafeteria	N/A	Caloric content of purchases	Point of sale system	8. Caloric content of purchases decreased^a^	Moderate
Cardenas et al. [69]	2015	Peru	Fruit was repositioned from a distant position to a more accessible location near the point of purchase. Additionally, signage highlighted health benefits of fruit consumption.	Pre-post	150 customers	> 1 week & ≤ 1 month	University cafeteria	N/A	Fruit purchases	Hand-counts	9. Fruit purchases were not affected	Moderate
Thorndike et al. [70]	2012	USA	Foods were labelled red, yellow and green. Additionally, healthy foods were located to convenient positions and unhealthy options were made less convenient.	Pre-post	1 cafeteria	1 < month(s) ≤ 6	Hospital cafeteria	N/A	Purchases of green items Purchases of red items	Point of sale system	10a. Purchases of green items increased^∞^. 10b. Purchases of red items decreased^∞^	Moderate
Steenhuis et al. [33]	2004	The Netherlands	In the food supply program, the availability of low-fat items increased and was made salient with signage.	RCT	17 cafeterias were randomly assigned to either of 4 conditions (including control and food supply program)	1 < month(s) ≤ 6	Worksite cafeteria	2% low educational level	Purchases of low-fat items	Point of sale system and questionnaire	11. Purchases of low-fat items were not affected	Weak
Thorndike et al. [71]	2014	USA	Items were labelled green, yellow or red. Additionally, items were rearranged to make some of the green items more visible and some red items less visible.	Pre-post	1 cafeteria	> 1 year	Hospital cafeteria	29% low educated jobs (service workers, administrative support, technicians)	Purchases of green items Purchases of red items	Point of sale system	12a. Purchases of green-labelled items increased^a^ 12b. Purchases of red-labelled items decreased^a^	Weak
Seward et al. [72]	2016	USA	The full intervention included traffic-light labels, accessibility changes and tray stickers visualizing recommended portions of food types. The minimal intervention only included accessibility changes.	CT	4 experimental cafeterias; 2 control cafeterias	1 < month(s) ≤ 6	University cafeteria	N/A	Purchases of green items Purchases of red items	Observer reported	13a. Purchases of green items were not affected in neither full or minimal intervention cafeterias. 13b. Purchases of red items were not affected in neither full or minimal intervention cafeterias.	Moderate
Lee-Kwan et al. [73]	2015	USA	During phase 1, menus were revised to emphasize healthy foods with labels. Consecutively, during phase 2, (additional) healthy sides and beverages were introduced and promoted.	CT	3 intervention stores; 4 control stores	1 < month(s) ≤ 6	Small food store	Carry-outs were based in low-income neighbourhoods	Purchases of healthy items	Point of sale system	14. Purchases of healthy items were not affected.	Moderate
*Availability, size, functionality, and presentation nudges*												
Diliberti et al. [74]	2004	USA	During baseline conditions, the portion size of the entree was the standard 100% portion; in the experimental condition the size was increased to 150%.	CT	180 customers	> 1 week & ≤ 1 month	Cafeteria	N/A	Energy intake	Food weighing	Energy intake from the pasta entree, accompaniments, and entire meal increased^a^	Moderate
Vandenbroele et al. [75]	2018	Belgium	Different sizes of sausages were available: a 150 g portion (default); a 125 g in-between portion; or a small, 100 g portion.	CT	161 customers who bought targeted product	> 1 week & ≤ 1 month	Supermarket	N/A	Meat purchases	Point-of-sale system	The introduction of smaller portion size alternatives was associated with less meat being purchased^a^	Moderate
Payne et al., study 1 [76]	2016	USA	Large green arrows were placed on the floor directing attention to the store’s produce section.	CT	1 intervention store; 1 control store	> 1 week & ≤ 1 month	Supermarket	N/A	Purchases of fruit & vegetables	Point-of-sale system	Green arrows on floors were associated with increased fruit and vegetable purchases^a^	Strong
Payne et al., study 2 [76]	2016	USA	Large green arrows were placed on the floor directing attention to the store’s produce section.	CT	1 intervention store; 1 control store	> 1 week & ≤ 1 month	Supermarket	N/A	Purchases of fruit & vegetables	Point-of-sale system	Green arrows on floors were associated with increased fruit and vegetable purchases^a^	Strong
Tal et al. [77]	2015	USA	Samples (no, apple, or cookie) were offered to participants at the entrance of the store	CT	120 customers	N/A	Supermarket	N/A	Purchases fruit & vegetables	Observer-reported	Receiving an apple sample was associated with increased subsequent purchases of fruits and vegetables vs. cookie or no sample (p = 0.06).	Weak

^a^Asterix indicates statistical significance (*p* < 0.05); ^∞^ Main findings are aggregated across two consecutive intervention phases for which data on statistical significance was not available

We were not able to visuzalize nine studies in harvest plots, due to outcomes that were difficult to categorize on relative healthiness (e.g., targeted foods for which insufficient information was available to determine this); the absence of formal statistical analysis or the use of a factorial design. These studies can be found in Additional file 3.

## Results

**Fig. 1:**
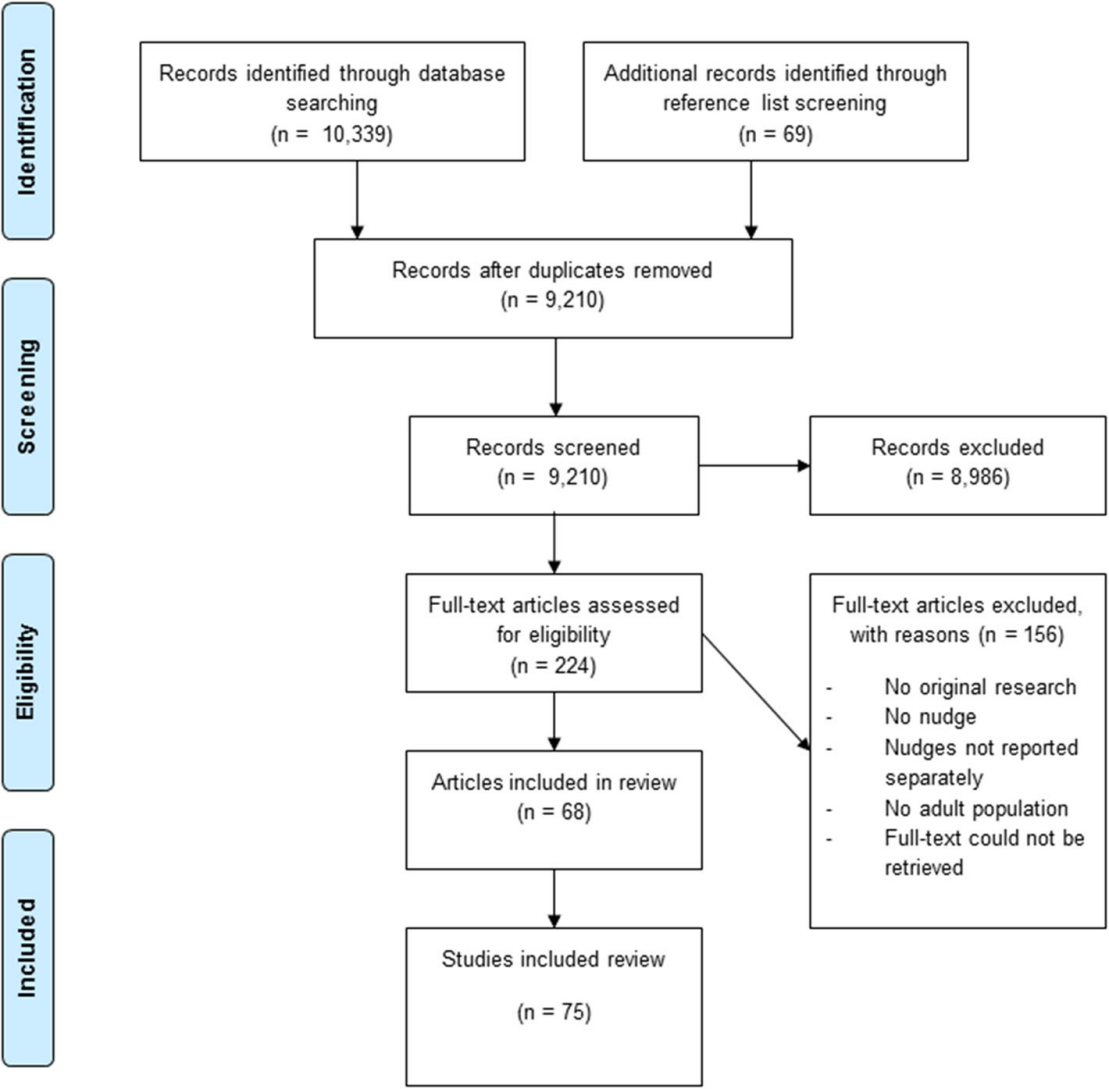
PRISMA flowchart of study inclusion

From the 9210 references identified from the database searches and reference list screening, 224 were eligible for full-text review, and 68 references were included in the narrative synthesis of findings. The 68 references comprised 75 studies (Fig. 1).

### Descriptive characteristics of included studies

Of the 75 retrieved studies, 42 studies were categorized as studying information nudges, ten studies were categorized as studying position nudges, 18 studies were categorized as studying mixed nudging interventions, two studies were categorized as studying size nudges, two studies were categorized as studying a functionality nudge, and one study was categorized as studying a presentation nudge. No studies were categorized as studying default or availability nudges. Given the vast amount of information nudges identified, we further categorized these groups of interventions into the following categories: information nudges using symbols (*n* = 15); information nudges providing nutrition information (*n* = 13); and information nudges using signage (*n* = 14). Studies most often employed a before-after design (56%), followed by a controlled trial design (32%) and randomized controlled trial design (12%). Only 19% of studies had an intervention duration longer than 6 months, and studies were most often situated in cafeterias (55%), followed by supermarkets (25%) and small food stores (16%).

### Effects of nudging by TIPPME category

#### Information nudges using symbols

**Fig. 2:**
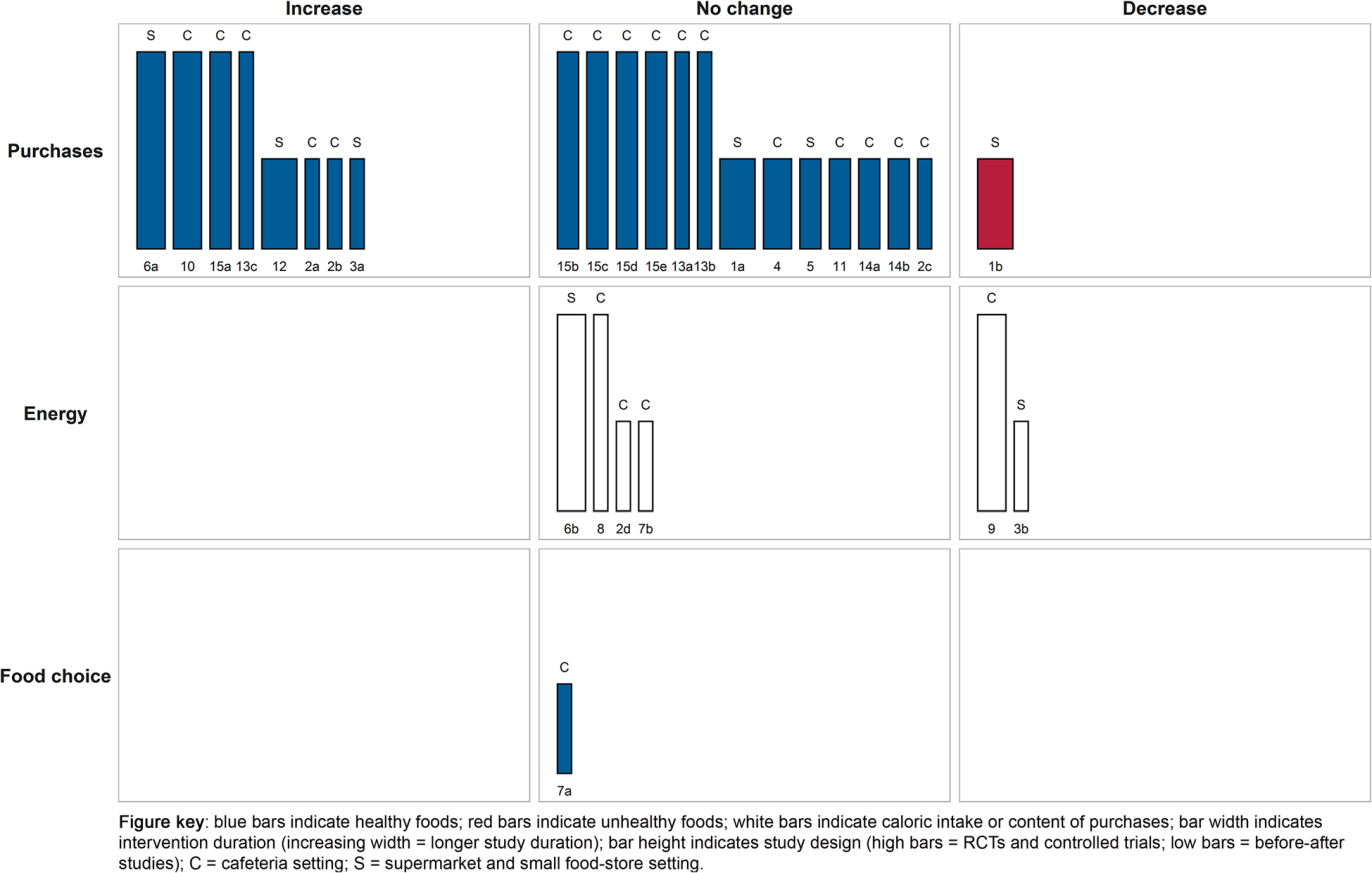
Harvest plot for information nudges using symbols

The harvest plot for information nudges using symbols is shown in Fig. 2 and study characteristics and main findings are presented in Table 2. Eight studies received a moderate quality rating, four received a weak quality rating, and three received a strong quality rating. Studies examining information nudges via symbols generally highlighted healthy or unhealthy foods using symbols such as star-ratings and promotional logos. The effects of information nudges using symbols were most often studied in association to purchasing outcomes. Overall, in mainly cafeteria settings, identifying healthy food items through the use of symbols generally did not affect purchases of those items [**1a, 2c, 4, 5, 11, 13a, 13b, 14a, 14b, 15b, 15c, 15d, 15e**], caloric content of purchases or caloric intake [**2d, 6b, 7b, 8**], or healthier food choice [**7a**]. Contrary, some other studies conducted in supermarket and cafeteria settings showed increased purchases of healthy foods and decreased purchases of unhealthy foods [**1b, 2a, 2b, 3a, 6a, 10, 12, 13c, 15a**] and decreased energy intake or content of purchases [**3b, 9**]. Concluding, the effects of highlighting healthy and unhealthy foods through the use of symbols in supermarket, small food store, and cafeteria settings were heterogeneous but showed a modest tendency towards no effects on studied outcomes.

#### Information nudges providing nutrition information

**Fig. 3:**
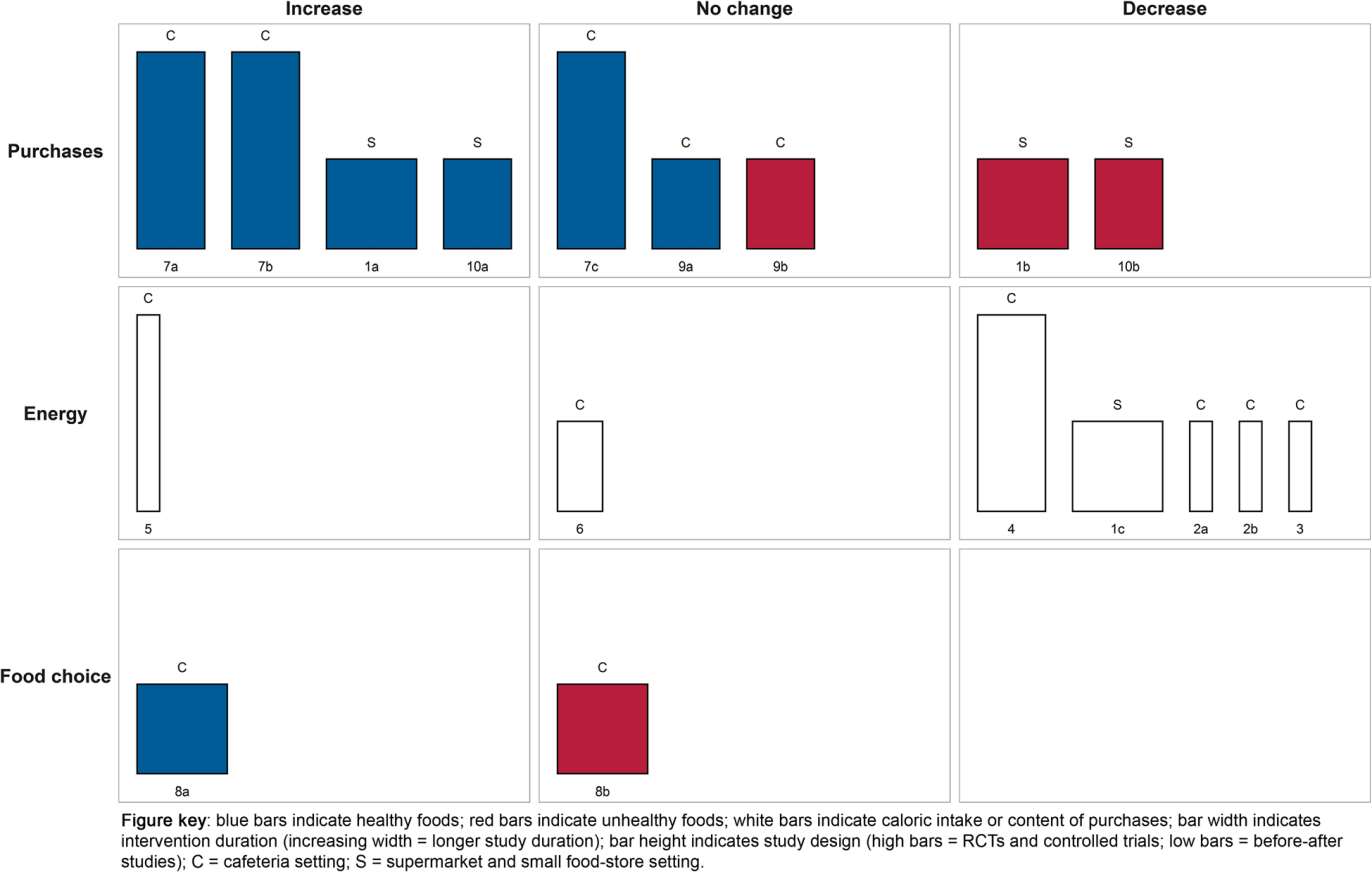
Harvest plot for information nudges providing nutrition information

The harvest plot of information nudges providing nutrition information is shown in Fig. 3 and study characteristics and main findings are presented in Table 2. Three studies could not be visualized in the harvest plots and are presented in Additional file 3. Seven studies received a moderate quality rating, five studies received a weak quality rating, and one study received a strong quality rating. Studies examining information nudges providing nutrition information usually did so by providing nutritional labels at the point-of-choice. The effects of nutrition information nudges were most often studied in relation to purchases as the outcome as well as energy intake or energy content of purchases. Some studies provide evidence that the provision of nutrition information in food purchasing environments increases purchases of or choice for healthy items [**1a, 7a, 7b, 8a, 10a**], decreases purchases of unhealthy items [**1b, 10b**], and similarly, decreases energy intake or energy content of purchases [**1c, 2a, 2b, 3, 4**], although one study observed increased energy intake [**5**]. Contrary, other studies found no effects on purchases of healthy or unhealthy items [**7c, 9a, 9b**], or on energy intake or content of purchases [**6**] or food choice [**8b**]. Concluding, the effects of providing nutrition information in supermarket, small food store and cafeteria settings were heterogeneous but showed a modest tendency towards beneficial effects on studied outcomes.

#### Information nudges using signage

**Fig. 4:**
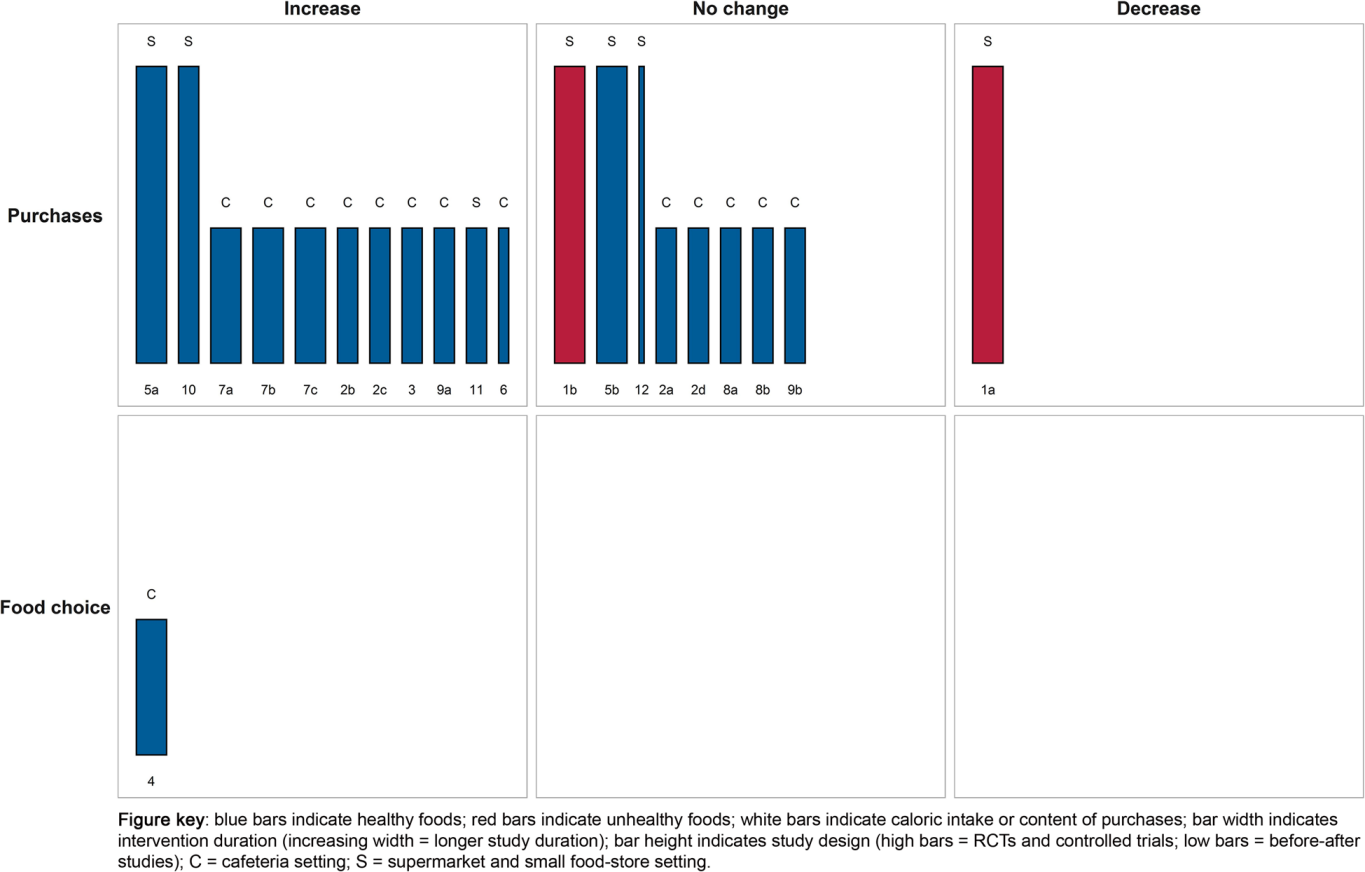
Harvest plot for information nudges using signage

The harvest plot of information nudges using signage is shown in Fig. 4 and study characteristics and main findings are presented in Table 2. Two studies could not be visualized in the harvest plots and are presented in Additional file 3. Eight studies received a moderate quality rating, three studies received a weak quality rating, and three studies received a strong quality rating. Studies examining information nudges using signage generally displayed posters with health prompts, social norms, or health primes. The effects of signage nudges were generally evaluated on purchasing outcomes and studies were primarily conducted within cafeteria settings. Signage was associated with increased purchases of healthy items in several studies [**2b, 2c, 3, 5a, 6, 7a, 7b, 7c, 9a, 10, 11**], increased choice for healthy food [**4**] and with decreased purchases of unhealthy items [**1a**]. Contrary, also no change in purchases of healthy or unhealthy [**1b, 2a, 2d, 5b, 8a, 8b, 9b, 12**] items were observed. Concluding, effects for information nudges using signage in supermarket, small food store, and cafeteria settings were heterogeneous but showed a modest tendency towards beneficial effects on studied outcomes.

#### Position nudges

**Fig. 5:**
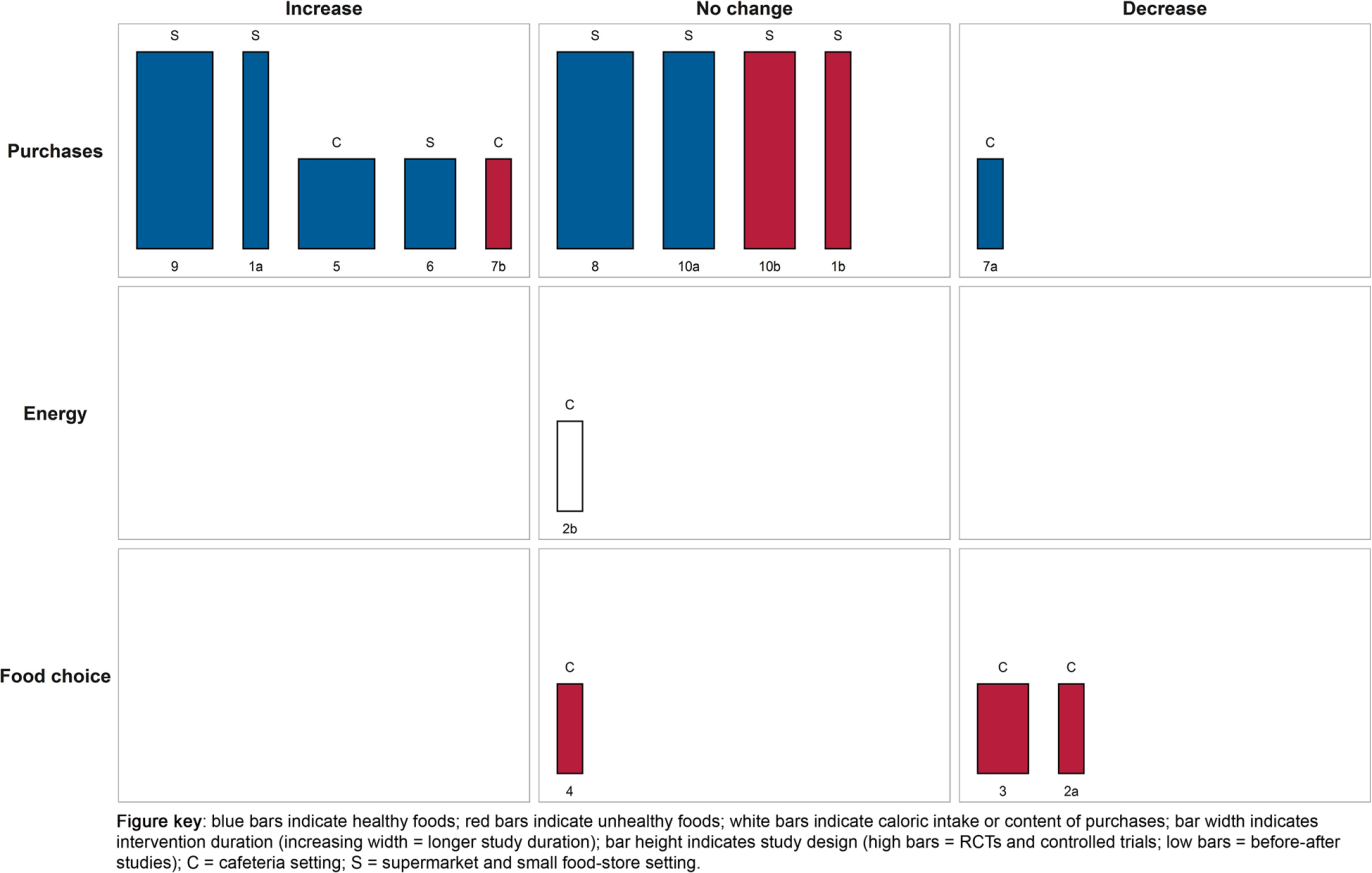
Harvest plot for position nudges

The harvest plot for position nudges is shown in Fig. 5 and study characteristics and main findings are presented in Table 2. Eight studies received a moderate quality rating and two received a weak quality rating. Studies examining position nudges generally manipulated proximity to healthy and unhealthy foods (e.g., decreasing proximity to healthy foods and increasing proximity to unhealthy foods). The effects of position nudges were most often studied in relation to purchasing outcomes. Overall, it can be concluded that in small food stores and cafeterias, increasing or decreasing the accessibility or visibility of healthy and unhealthy foods, respectively, showed increased purchases of healthy foods and decreased choice for unhealthy foods [**1a, 2a, 3, 5, 6, 9**]. However, other studies conducted in larger purchasing contexts such as supermarkets showed no effects on healthy food purchases **[8, 10a].** Moreover, purchases of relocated unhealthy snacks (e.g., snacks that were relocated to more distant locations as a consequence of making healthy foods more accessible) [**1b, 10b**], energy intake [**2b**], or food choice [**4**] were not affected in both small and larger purchasing contexts. Lastly, one study showed counterintuitive findings, with increased and decreased purchases of unhealthy and healthy items, respectively, when healthy items had been made more accessible [**7a, 7b**]. Concluding, the effects of altering the proximity of healthy and unhealthy foods showed a modest tendency towards beneficial effects on outcomes in primarily smaller food purchasing environments, but not in larger food purchasing environments.

#### Mixed nudging interventions

**Fig. 6:**
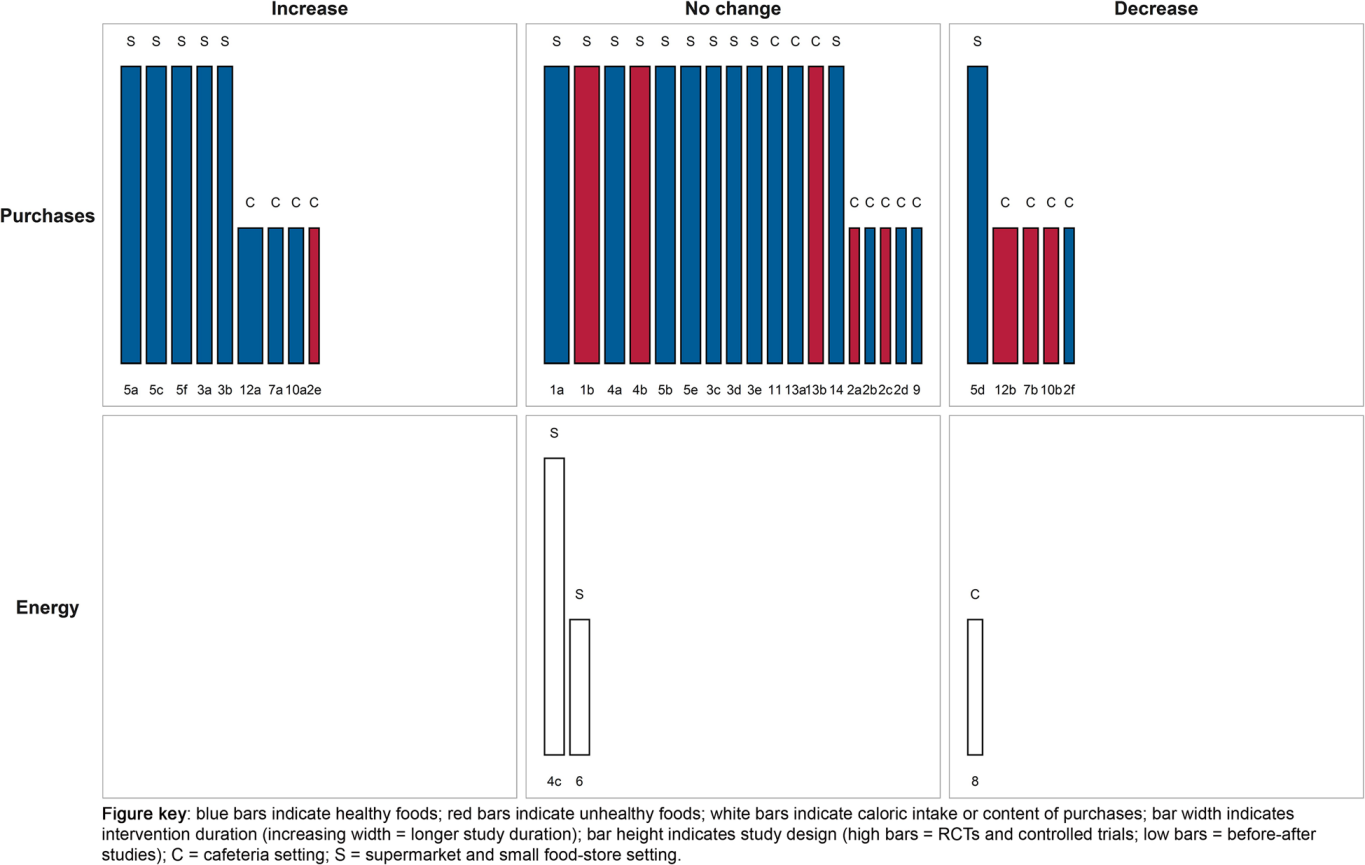
Harvest plot for mixed nudging interventions

Several studies were identified that studied a combination of TIPPME intervention categories, which we phrased ‘mixed nudging interventions’. The harvest plot for mixed nudging intervention is shown in Fig. 6 and study characteristics and main findings are presented in Table 2. Four studies could not be visualized in the harvest plots and are presented in Additional file 3.

Eight studies received a moderate quality rating, eight studies received a weak quality rating, and two studies received a strong quality rating. The effects of mixed intervention nudges were most often studied in relation to purchasing outcomes in cafeteria or supermarket settings. Moreover, studies were often characterized by high quality study designs (e.g., RCTs and controlled trials). As for the effects of mixed nudging interventions on the outcomes studied, mixed nudging interventions generally did not affect purchases of healthy items [**1a, 2b, 2d, 3c, 3d, 3e, 4a, 5b, 5e, 9, 11, 13a, 14**] or unhealthy items [**1b, 2a, 2c, 4b, 13b**], or energy intake or -content of purchases [**4c, 6**]. Contrary, some studies observed increased purchases of healthier items [**3a, 3b, 5a, 5c, 5f, 7a, 10a, 12a**], decreased purchases of unhealthy items [**7b, 10b, 12b**], and decreased calorie content of purchases [**8**]. Also some counterintuitive findings were observed, with mixed nudging interventions being associated with increased purchases of unhealthy items [**2e**] and decreased purchases of healthy items [**2f, 5d**]. Concluding, the effects mixed nudging interventions in supermarket, small food store, and cafeteria settings were heterogeneous but showed a modest tendency towards no changes in studied outcomes.

#### Availability, size, functionality, and presentation nudges

Two studies were categorized as size nudges [74, 75]. In these studies, increasing the portion size of an entrée [74] and decreasing the portion size of sausages [75], was associated with increased energy intake and decreased meat purchases, respectively. Two studies described the effects of a functionality nudge [76]. In these studies, arrows on supermarket floors indicating the location of fresh fruits and vegetables were associated with increased fruit and vegetable purchasing. One study was categorized as a presentation nudge, during which participants were provided with a healthy or unhealthy sample and subsequent purchases in a supermarket were monitored [77]. The study showed that the consumption of a healthy sample was associated with increased subsequent healthy purchases.

### Evidence for differential effects across SEP

Six studies evaluated the effects of nudges across levels of SEP, for which several indicators were used including educational level, food security, job type, and income. In subgroup analyses, there were modest indications that nudges – including signage, mixed nudging interventions, and position nudges – were significantly more effective among people with a lower educational level [44], in people with food insecurity [63], or in people on a food assistance program [59], respectively. Similarly, in two other mixed nudging intervention studies which used traffic-light labelling and accessibility changes, the extent to which red and green-labelled purchases were affected by the intervention differed in magnitude across job type in subgroup analyses [71] and the effect of the intervention on red-labelled purchases was significantly modified by job type, but not for overall purchases [67]. However, no evident pattern in purchasing differences across job types could be discerned, as job types could not be clearly classified by SEP. In another study which examined the effect of an information nudge providing nutrition information on calorie intake, no significant effect modification by income or educational level was observed [37].

## Discussion

### Main findings

In the present review, we aimed to assess the evidence for the effectiveness of nudges as classified according to TIPPME in promoting healthy purchases, food choice, or affecting energy content of purchases or intake within real-life food purchasing environments. Additionally, we aimed to investigate whether the effects of nudges are moderated by SEP. We observed that the evidence to date predominately focused on the effectiveness of information nudges (56%) and position nudges (13%), while less evidence is available on the effectiveness of other types of TIPPME nudging interventions. We also observed that studies often investigated short-term outcomes, with 81% of studies having an intervention duration shorter than 6 months. Also, the studies often relied on non-randomized designs and were most often conducted in cafeteria or supermarket settings.

The harvest plots showed that for information and position nudges modest tendencies towards beneficial effects on studied outcomes were present. Finally, we found indications that the effects of nudges may be moderated by SEP, showing larger effects among low SEP individuals. However, evidence was limited in quantity and the use of different measures of SEP hampered comparison of the evidence. Overall, studies were generally considered of moderate or weak quality, raising concerns about potential bias and warranting caution in the interpretation of the results.

Findings from the present review are in line with previous literature. Similar to the present study, a scoping review conducted by Hollands et al. concluded that most studies focused on information nudges [78]. The effectiveness of information nudges is however debated, as they deviate from the original definition of nudging, by relying partly on cognitive processing. One previous meta-analysis of field studies by Cadario and Chandon explored the effectiveness of nudges, using their own categorization of cognitive nudges, affective nudges and behavioural nudges. They concluded that cognitive nudges were least effective in affecting selection and consumption outcomes [79], observing a small effect size of d = 0.12, supporting the argument that information nudges are ‘sub-optimal’. In the present review, we observed that information nudges – largely overlapping with the definition of cognitive nudges by Cadario and Chandon – positively affected outcomes, but we could not compare the magnitude of effects to other TIPPME nudges given the inability to meta-analyse findings. Further evidence that information nudges work, even though considered ‘sub-optimal’ in terms of how they operate on a psychological level, comes from two recent systematic reviews and meta-analyses of nutritional package and/or point-of-purchase labelling in primarily supermarkets, cafeterias, and restaurants, showing statistically significant average decreases of 6.6 and 7.8% in energy intake, respectively [80, 81], although for the latter review the quality of evidence was rated as low.

We also observed a tendency towards healthier purchasing in smaller food purchasing contexts for position nudges. Although evidence is tentative and qualitative in nature, this finding is in line with multiple systematic reviews that examined the effects of position nudges on consumption and selection; choice, sales or servings; or on sales and consumption in primarily laboratory settings [13], school settings [11], and a range of micro-environments including cafeteria and laboratory settings [12], respectively. However, all reviews highlight that effects are generally small in magnitude and that the quality of evidence is considered to be low.

Finally, we observed that the effects of nudges may differ by SEP, with limited studies observing somewhat stronger effects in low SEP populations. Only one other systematic review and meta-analysis that examined the effectiveness of availability and proximity nudges systematically assessed whether the effects of these interventions were potentially modified by SEP, and found that effect sizes for position nudges were larger among studies conducted among populations with low deprivation status, as compared to studies conducted among populations from both high and low deprivation status [13]. For availability nudges, insufficient data was available to assess whether intervention effects were modified by SEP. An important reason for why evidence is limited in the present review, may be due to the fact that it is challenging to obtain detailed information on SEP in studies conducted in real-life food purchasing environments, as there is often less active engagement with the research population. For example, studies often monitor purchases following a nudging intervention, without consent or active participation of customers.

### Strengths and limitations

Some limitations of the present review need to be addressed. First, given the substantial heterogeneity in study characteristics and incomplete study reporting, it was not possible to quantify the effects of the TIPPME intervention types using conventional meta-analyses techniques. An important reason for the heterogeneous study characteristics and study findings may relate to the focus on real-life purchasing contexts which are naturally less controlled environments as compared to laboratory settings. Additionally it may be due to our studied outcomes which were heterogeneous in terms of the types of foods that were targeted with nudging strategies. However, the use of harvest plots offers a visually appealing way to summarize the study information and study findings. This approach is preferable over a narrative analysis of study findings, as information is more easily digested by the reader and also less prone to bias, as studies are plotted in a systematic way [82]. Second, very few studies assessed dietary intake as outcome of nudging interventions. Alternatively, energy content of purchases was often calculated as a proxy of energy intake. Therefore, the majority of evidence is based on the evaluation of food purchases. As nudging is often suggested as a potentially important strategy in battling the obesity epidemic, it is crucial to evaluate its effects on more proximal health parameters, such as dietary intake, as well. Third, we adopted a broad search strategy, including general nudging terms (e.g., nudging and choice architecture) as well as more specific nudging terms (e.g., signage) according the TIPPME typology. As a result of this search strategy, studies were included that did not clearly indicate to test a nudge, but did comply with nudging definitions laid out by the TIPPME typology. As these studies provided little theoretical background of the intervention under study, there was often limited information available to categorize the study according to TIPPME. For example, studies we categorized as information nudges based on the TIPPME definition, may partly rely on cognitive processing, and therefore may not satisfy the criteria for nudging. Finally, the majority of studies received a moderate to weak quality rating. Major quality issues related to the study design, which was often not randomized, which consequently raised concerns about potential for confounding. Concerns about the quality of nudging studies have also been highlighted in previous reviews [11–13].

Strengths of the current review include that it used an extensive search strategy, not only using ‘nudging’ and ‘choice architecture’ as search terms, but adding specific nudging intervention types as search terms as well. Indeed, a previous systematic review investigating the effectiveness of nudging strategies only included studies if they were specified as such by the original authors, resulting in only thirteen eligible publications [83]. Additionally, the present review builds upon the TIPPME typology which was the result of an extensive scoping review, and therefore provides a useful conceptual framework for structuring the evidence base. However, we acknowledge that categorizations remain broad and may be susceptible to different interpretations, and further enhancement of conceptual clarity is needed.

### Implications for improved methods

Given the limitations of the evidence base addressed in this review, we provide several suggestions for improved methods. First, given the level of heterogeneity in study characteristics there is an urgent need for harmonization of methods in nudging studies to facilitate evidence accumulation. It is therefore important to establish common measures to asses SEP, such as composite measures combining both income, education, and job status [84]. Additionally, adherence to reporting standards such as Journal Article Reporting Standards (JARS) as laid out by the American Psychological Association would improve study reporting and therefore enhance evidence synthesis. Moreover, the field of psychological and behavioural science has been scrutinized for its inability to replicate some of its findings [85]. For example, a recent pre-registered study found no association between plate size and food consumption, which contrasted with earlier findings [86]. Therefore, efforts such as pre-registration of study protocols which allow replication are warranted to further advance the field of (nutrition) nudging [87].

### Implications for future research and practice

From the present evidence, we highlight the following knowledge gaps present in nudging literature. First, future studies should focus on studying the effectiveness of non-information nudges (e.g., availability, position, functionality, or sizing nudges) in real-world settings. Second, given the limited available data on potential moderators of nudging effectiveness in real-world settings, the use of loyalty cards containing customer’s personal information would be a valuable contribution to the existing literature, allowing to examine the role of potential moderators such as age, sex, and SEP. Third, nudging studies often only targeted limited food categories, which does not justify complex food environments in which multiple other food choices are made. Moreover, it is difficult to make inferences about what changes in purchases of a selected number of foods actually constitutes in terms of an individual’s health. Therefore, future nudging studies that use loyalty cards, could nudge a wider array of food products and estimate changes in overall dietary quality on an individual level. Fourth, as the included literature in the present study mainly studied short-term effects, future studies should consider including a longer follow-up, as this long-term effectiveness is crucial to assess potential public health impact. Lastly, the present review highlights the viability of conducting nudging interventions in real-life purchasing contexts. Consequently, local policy makers or owners of local food stores could be encouraged to implement nudging interventions at local level. From a policy perspective, it is also of importance to consider the ethical aspects of nudging, which have been outlined previously [88].

## Conclusion

This systematic review was the first to examine the effectiveness of nudging interventions on purchases, energy intake or content of purchases, and food choice in real-life food purchasing environments, using an elaborate search strategy drawing upon the TIPPME framework. We showed that evidence mainly focuses information and position nudges, while less evidence is available on the effectiveness of other TIPPME intervention types. We qualitatively demonstrated that information and position nudges might be effective in improving outcomes, especially purchasing outcomes, and that SEP may be a moderator for the effectiveness of nudges. However, evidence is limited and difficult to compare. More high-quality studies focusing on non-information nudges and examining long-term effectiveness in real-life food purchasing environments and obtaining detailed data on participant’s SEP are needed.

## Supplementary information

**Additional file 1.** PRISMA checklist.

**Additional file 2.** Search strategy for bibliographic databases.

**Additional file 3.** Studies not appropriate for visualisation in harvest plots.

## Data Availability

The dataset supporting the conclusions of this article is included within the article.
