# Supplementary material for: The effects of nudges on purchases, food choice, and energy intake or content of purchases in real-life food purchasing environments: a systematic review and evidence synthesis
Source: Nutr J. 2020 Sep 17;19:103. doi: 10.1186/s12937-020-00623-y (PMC7500553; doi:10.1186/s12937-020-00623-y)
Supplement: Supplementary file 2 — Additional file 2. Search strategy for bibliographic databases. [file 12937_2020_623_MOESM2_ESM.docx]

**Additional file 2** Search strategy for bibliographic databases

PubMed

(nudg*[Title/Abstract] OR choice architect*[Title/Abstract] OR environmental intervention*[Title/Abstract] OR environmental change*[Title/Abstract] OR environmental cue*[Title/Abstract] OR behavioral economic*[Title/Abstract] OR behavioural economic*[Title/Abstract] OR heuristic*[Title/Abstract] OR availability[Title/Abstract] OR assortment[Title/Abstract] OR variety[Title/Abstract] OR position[Title/Abstract] OR placement[Title/Abstract] OR proximity[Title/Abstract] OR visibility[Title/Abstract] OR salience[Title/Abstract] OR accessibility[Title/Abstract] OR convenience[Title/Abstract] OR trolley[Title/Abstract] OR cart[Title/Abstract] OR tray[Title/Abstract] OR presentation[Title/Abstract] OR packaging[Title/Abstract] OR portion size[Title/Abstract] OR unit size[Title/Abstract] OR package size[Title/Abstract] OR nutrition information[Title/Abstract] OR health information[Title/Abstract] OR label*[Title/Abstract] OR prompt*[Title/Abstract] OR social norm*[Title/Abstract] OR reminder*[Title/Abstract] OR feedback[Title/Abstract] OR default[Title/Abstract]) AND (diet[Title/Abstract] OR dietary[Title/Abstract] OR food*[Title/Abstract] OR intake[Title/Abstract] OR purchas*[Title/Abstract] OR meal*[Title/Abstract] OR calori*[Title/Abstract] OR consum*[Title/Abstract] OR spend*[Title/Abstract] OR choice*[Title/Abstract] OR sale*[Title/Abstract]) AND (store[Title/Abstract] OR shop[Title/Abstract] OR supermarket[Title/Abstract] OR market[Title/Abstract] OR retail*[Title/Abstract] OR lunchroom[Title/Abstract] OR cafeteria[Title/Abstract] OR canteen[Title/Abstract] OR kiosk[Title/Abstract] OR point of purchase[Title/Abstract] OR fast-food outlet[Title/Abstract] OR fast-food restaurant[Title/Abstract] OR food-service operat*[Title/Abstract]) NOT (animals[mh] NOT humans[mh])

Filters: Journal Article; English; Dutch

Embase

('nudg*':ab,ti OR 'choice architect*':ab,ti OR 'environmental intervention*':ab,ti OR 'environmental change*':ab,ti OR 'environmental cue*':ab,ti OR 'behavioral economic*':ab,ti OR 'behavioural economic*':ab,ti OR 'heuristic*':ab,ti OR 'availability':ab,ti OR 'assortment':ab,ti OR 'variety':ab,ti OR 'position':ab,ti OR 'placement':ab,ti OR 'proximity':ab,ti OR 'visibility':ab,ti OR 'salience':ab,ti OR 'accessibility':ab,ti OR 'convenience':ab,ti OR 'trolley':ab,ti OR 'cart':ab,ti OR 'tray':ab,ti OR 'presentation':ab,ti OR 'packaging':ab,ti OR 'portion size':ab,ti OR 'unit size':ab,ti OR 'package size':ab,ti OR 'nutrition information':ab,ti OR 'health information':ab,ti OR 'label*':ab,ti OR 'prompt*':ab,ti OR 'social norm*':ab,ti OR 'reminder*':ab,ti OR 'feedback':ab,ti OR 'default':ab,ti) AND ('diet':ab,ti OR 'dietary':ab,ti OR 'food*':ab,ti OR 'intake':ab,ti OR 'purchas*':ab,ti OR 'meal*':ab,ti OR 'calori*':ab,ti OR 'consum*':ab,ti OR 'spend*':ab,ti OR 'choice*':ab,ti OR 'sale*':ab,ti) AND ('store':ab,ti OR 'shop':ab,ti OR 'supermarket':ab,ti OR 'market':ab,ti OR 'retail*':ab,ti OR 'lunchroom':ab,ti OR 'cafeteria':ab,ti OR 'canteen':ab,ti OR 'kiosk':ab,ti OR 'point of purchase':ab,ti OR 'fast-food outlet':ab,ti OR 'fast-food restaurant':ab,ti OR 'food-service operat*':ab,ti) AND [embase]/lim NOT [medline]/lim NOT ([animals]/lim NOT [humans]/lim) AND 'article'/it

PsycINFO

(nudg* OR choice architect* OR environmental intervention* OR environmental change* OR environmental cue* OR behavioral economic* OR behavioural economic* OR heuristic* OR availability OR assortment OR variety OR position OR placement OR proximity OR visibility OR salience OR accessibility OR convenience OR trolley OR cart OR tray OR presentation OR packaging OR portion size OR unit size OR package size OR nutrition information OR health information OR label* OR prompt* OR social norm* OR reminder* OR feedback OR default).ab,ti. AND (diet OR dietary OR food* OR intake OR purchas* OR meal* OR calori* OR consum* OR spend* OR choice* OR sale*).ab,ti. AND (store OR shop OR supermarket OR market OR retail* OR lunchroom OR cafeteria OR canteen OR kiosk OR point of purchase OR fast-food outlet OR fast-food restaurant OR food-service operat*).ab,ti.
