# Supplementary material for: The effects of nudges on purchases, food choice, and energy intake or content of purchases in real-life food purchasing environments: a systematic review and evidence synthesis
Source: Nutr J. 2020 Sep 17;19:103. doi: 10.1186/s12937-020-00623-y (PMC7500553; doi:10.1186/s12937-020-00623-y)
Supplement: Supplementary file 3 — Additional file 3. Studies not appropriate for visualisation in harvest plots. [file 12937_2020_623_MOESM3_ESM.docx]

**Additional file 3** Studies not appropriate for visualisation in harvest plots

| **Author** | **Year** | **Type of nudge** | **Country** | **Nudge description** | **Study size** | **Study design** | **Intervention duration** | **Setting** | **Study outcome(s)** | **Outcome assessment** | **Main finding(s)** | **Quality assessment** | **Reason for not being included in harvest plot** |
| --- | --- | --- | --- | --- | --- | --- | --- | --- | --- | --- | --- | --- | --- |
| Roy *et al.* ^(1)^ | 2016 | Nutrition information | USA | Caloric information was displayed on menus and on laminated menus at the counter. | 1 food outlet | Pre-post | 1 < month(s) ≤ 6 | Small food store | Purchases of menu items | Point of sale system | Calorie labelling was associated with increased purchases of one relatively low-calorie menu option* and decreased purchases of the highest calorie menu-option* | Moderate | Outcome difficult to categorize (healthy vs. unhealthy) |
| Sacks *et al.* ^(2)^ | 2009 | Nutrition information | UK | Products were labelled with traffic-light labels | Supermarkets across the UK | Pre-post | > 1 week & ≤ 1 month | Supermarket | Purchases of targeted products | Point of sale system | Labelling was associated with increased sales of ready meals*; but these were not more healthy. The sales of sandwiches were unaffected. | Moderate | Outcome difficult to categorize (healthy vs. unhealthy) |
| Cinciripini *et al.* ^(3)^ | 1984 | Nutrition information | USA | The caloric value of menu items was displayed on large tripods (caloric feedback). Subsequently, healthy foods were identified with a green triangle (labelling). | 5,542 observations | Pre-post | 1 < month(s) ≤ 6 (both interventions) | University cafeteria | Purchases of cafeteria items | Observer reported | Caloric feedback was associated with decreased purchases of red meat, poultry/fish, carbohydrates, and dairy; and increased purchases of fruit and vegetables, low-fat dairy and salad. Labelling was associated with increased purchases of high-fat desserts and red meat, and decreased purchases of salads, dairy and carbohydrates. | Weak | Outcome difficult to categorize (healthy vs. unhealthy) |
| Papies *et al.* ^(4)^ | 2010 | Signage | The Netherlands | A poster announcing a recipe for a 'good and slim figure' was manipulated (poster present/absent) in a butcher while participants were exposed to attractive food cues (grilled chicken samples). | 156 customers | CT | N/A | Butcher | Selection of grilled chicken | Observer-reported | The healthy recipe decreased selection of grilled chicken among restrained but not unrestrained eaters* | Weak | Factorial design |
| Wagner *et al.* ^(5)^ | 1988 | Signage | USA | Messages with ‘health’ prompts were placed at several locations in the restaurant | 1 intervention store; 1 control store | CT | > 1 week & ≤ 1 month | Fast food restaurant | Salad bar purchases | Point of sale system | Salad purchases increased | Weak | No formal statistical analysis |
| Sigurdsson *et al.* ^(6)^ | 2014 | Mixed nudging intervention | Norway | Confectionary at the checkout was replaced by healthy foods. Additionally, advertisement was added, reinforcing the healthiness of the healthy check-out foods. | 1 intervention store; 1 control store | Pre-post | > 1 week & ≤ 1 month | Supermarket | Purchases of healthy check-out foods  Purchases of relocated unhealthy foods | Point of sale system | Purchases of healthy targeted check-out foods increased; purchases of relocated unhealthy products decreased | Moderate | No formal statistical analysis |
| Wisdom *et al.* ^(7)^ | 2010 | Mixed nudging intervention | USA | The menus varied in a 2 (daily calorie recommendation offered or not) × 2 (calorie information for menu items shown or not) × 3 (convenience of healthy options) design | 638 participants | CT | N/A | Fast food restaurant | Total calorie intake | Questionnaire | Provision of calorie information* and calorie recommendations* was associated with decreased calorie intake; grouping healthy menu options together also decreased total calorie intake* | Weak | Factorial design |
| Rushakoff *et al.* ^(8)^ | 2017 | Mixed nudging intervention | USA | Environmental changes included inventory changes, installation of point-of-choice materials (shelf strips and cookbook), taste tests, and display improvements. | n=233 at baseline; n=211 at follow-up | Pre-post | > 1 year | Small food store | Purchases and reported intake of foods | Questionnaire | Increased purchasing and consumption were reported for some healthy items (no statistical testing). | Weak | No formal statistical analysis |
| Van Kleef *et al.*, study 2 ^(9)^ | 2012 | Mixed nudging intervention | The Netherlands | The shelf arrangement of healthy snacks (healthy snacks on top vs. bottom shelves) and the assortment structure (75% healthy snacks vs. 25% healthy snacks) was manipulated. | 291 purchases | RCT | > 1 week & ≤ 1 month | Hospital cafeteria | Sales of healthy and unhealthy snacks | Hand-counts | Predominantly healthy snack assortment structure was associated with increased purchases of healthy snacks*; shelf arrangement did not impact on healthy snack purchases. | Weak | Factorial design |

*p<0.05

**References**

1. Roy R, Beattie-Bowers J, Ang SM *et al.* (2016) The Effect of Energy Labelling on Menus and a Social Marketing Campaign on Food-Purchasing Behaviours of University Students. *BMC Public Health* 16, 727.

2. Sacks G, Rayner M, Swinburn B (2009) Impact of front-of-pack 'traffic-light' nutrition labelling on consumer food purchases in the UK. *Health Promot Int* 24, 344-352.

3. Cinciripini PM (1984) Changing food selections in a public cafeteria: An applied behavior analysis. *Behav Modif* 8, 520-539.

4. Papies EK, Hamstra P (2010) Goal priming and eating behavior: enhancing self-regulation by environmental cues. *Health Psychol* 29, 384-388.

5. Wagner JL, Winett RA (1988) Prompting one low-fat, high-fiber selection in a fast-food restaurant. *J Appl Behav Anal* 21, 179-185.

6. Sigurdsson V, Larsen NM, Gunnarsson D (2014) Healthy food products at the point of purchase: An in-store experimental analysis. *J Appl Behav Anal* 47, 151-154.

7. Wisdom J, Downs JS, Loewenstein G (2010) Promoting Healthy Choices: Information versus Convenience. *American Economic Journal: Applied Economics* 2, 164-178.

8. Rushakoff JA, Zoughbie DE, Bui N *et al.* (2017) Evaluation of Healthy2Go: A country store transformation project to improve the food environment and consumer choices in Appalachian Kentucky. *Prev Med Rep* 7, 187-192.

9. Van Kleef E, Otten K, van Trijp HC (2012) Healthy snacks at the checkout counter: a lab and field study on the impact of shelf arrangement and assortment structure on consumer choices. *BMC Public Health* 12, 1072.
